# Real Time SPR Assessment of the Structural Changes of Adaptive Dynamic Constitutional Frameworks as a New Route for Sensing

**DOI:** 10.3390/ma15020483

**Published:** 2022-01-09

**Authors:** Sorin David, Mihaela Gheorghiu, Sanaa Daakour, Raluca-Elena Munteanu, Cristina Polonschii, Szilveszter Gáspár, Mihail Barboiu, Eugen Gheorghiu

**Affiliations:** 1 International Centre of Biodynamics, 060101 Bucharest, Romania; sdavid@biodyn.ro (S.D.); mgheorghiu@biodyn.ro (M.G.); rmunteanu@biodyn.ro (R.-E.M.); cpolonschii@biodyn.ro (C.P.); sgaspar@biodyn.ro (S.G.); 2 Adaptive Supramolecular Nanosystems Group, Institut Europeen des Membranes, University of Montpellier, ENSCM-CNRS, 34095 Montpellier, France; sanaadaakour@hotmail.com

**Keywords:** dynamic constitutional frameworks, signal amplification by gold nanoparticles, conformational dynamics, surface plasmon resonance analysis, pH sensing

## Abstract

Cross linked gold-dynamic constitutional frameworks (DCFs) are functional materials of potential relevance for biosensing applications, given their adaptivity and high responsivity against various external stimuli (such as pH, temperature) or specific interactions with biomolecules (enzymes or DNA) via internal constitutional dynamics. However, characterization and assessment of their dynamic conformational changes in response to external stimuli has never been reported. This study proves the capability of Surface Plasmon Resonance (SPR) assays to analyse the adaptive structural modulation of a functional matrix encompassing 3D gold-dynamic constitutional frameworks (Au-DCFs) when exposed to pH variations, as external stimuli. We analyse Au-DCFs formed from Au nanoparticles, (AuNP) connected through constitutionally dynamic polymers, dynamers, with multiple functionalities. For increased generality of this proof-of-concept assay, Au-DCFs, involving DCFs designed from 1,3,5-benzene-tricarbaldehyde (BTA) connecting centres and polyethylene glycol (PEG) connectors, are covalently attached to standard SPR sensing chips (Au nanolayers, carboxyl terminated or with carboxymethyl dextran, CMD top-layer) and analysed using state-of-the art SPR instrumentation. The SPR effects of the distance from the Au-DCFs matrix to the Au nanolayer of the sensing chip, as well as of Au-DCFs thickness were investigated. This study reveals the SPR response, augmented by the AuNP, to the conformational change, i.e., shrinkage, of the dynamer and AuNP matrix when decreasing the pH, and provides an unexplored insight into the sensing applicability of SPR real-time analysis of adaptive functional materials.

## 1. Introduction

### 1.1. Dynamers and Their Analytical Potential

Linear dynamers and cross-linked 3D dynamic constitutional frameworks—DCFs— are dynamic polymers in which monomeric units and connection centres are associated through reversible covalent bonds [1]. Several reversible covalent reactions have been explored and the arrangement of dynamic networks has been diversified, providing adaptive systems with higher complexity and multiple responsiveness. They can be responsive to internal constitutional interactions, to various external stimuli, such as pH, temperature, pressure, etc. [2], or to specific interactions with biomolecules such as enzymes [3] or DNA [4]. These responses lead to systemic adaptation and associated changes in their physical and/or chemical properties. Some of the effective factors, especially pH and redox reactions, are often found in biological systems frameworks, which make the dynamic covalent materials suited to mimic bio-functional systems. They are capable of forming strong and resilient hydrogels, including networks of biological macromolecules which can take up large amounts of water while exhibiting ease of processing, self-healing, and antimicrobial or cell supporting features [2,3,4,5]. Their application in the biosensing field is further warranted given the versatile incorporation in their constitutional framework of metallic entities. Gold nanoparticles (AuNPs) are stable and biocompatible metallic entities with unique electrical, optical and surface chemical properties AuNPs characterized by strong adsorption ability, large specific surface area and formation of surface plasmon resonance band [6] were applied in various sensing formats (i.e., direct, sandwich or NP enhanced) involving bio-recognition mechanisms [7], catalysis and biosynthesis [8]. Their surface functionalization sheds light on a diverse collection of functional moieties that can be used for biomolecular interaction. Imine-based dynamic combinatorial chemistry was already applied on the surface of AuNP to (i) develop new receptors for either the simple recognition of biomacromolecules such as DNA [9], or for the complex one with self-selection of nucleotides in presence of ions [10] as well as (ii) to form molecular print boards suitable to modifications [11]. We previously demonstrated that imine-PEG1500 DCFs are stable in water for weeks in neutral and down to weak acidic pH > 3 conditions, while being hydrolysed in a strongly acidic aqueous solution at pH = 1 [12].

### 1.2. SPR in Sensing, in Particular for Conformational Changes

Surface Plasmon Resonance (SPR) is a very sensitive technique for measurements of minute changes in the refractive index of the complex media near a sensing surface with plasmonic features. Careful calibrations and appropriate choice of the affinity ligands allow the detection of very low concentrations of targeted analytes, as well as the highly accurate determination of binding kinetics. Some limitations of the SPR approaches reside in the intrinsic confinement of the sensitivity to depth regions of, at most, a few hundred nanometres from the sensing surface and to analytes with moderate/large molecular weight, as well as in the need to immobilize the ligand, without altering its structure, onto the sensing surface. Nevertheless, SPR is a well-established sensing technique, the gold standard for biomolecular interaction analysis. In a step forward, proof of concept reports highlight the potential sensitivity of SPR assays to conformational changes [13,14] undergone by affinity ligands decorating the sensing chips. SPR was used in the evaluation of conformational changes of sensitive layers (proteins, polymers) in response to modifications of the parameters of the surrounding media (e.g., pH, ionic strength, etc.) or for small analyte detection. Studies indicate that among novel SPR-powered sensing avenues: (a) the detection of low molecular analytes via refractive index changes due to analyte–ligand interaction within the sensing layer [14,15,16], and (b) it is possible to evaluate the presence and concentration of ions in solutions by measuring the conformational changes in a sensitive protein [13,17]. The influences of the physicochemical properties of the buffer media were analysed to investigate the alterations induced by the pH of solution to the functional layers of SPR sensing chips as well as to the immobilized protein [18]. Moreover, the use of gold nanoparticles in plasmonic modified probes (e.g., within the sensing protein layer [19] or in the functional polymer layer [20]) was advanced in SPR assays for signal amplification, supporting the analyses of minute, analyte-specific conformational changes in DCFs functionalised SPR chips.

### 1.3. Aim of the Study

As SPR has not been used to assess conformational/morphological changes in dynamers (aside investigation of the binding affinity [21,22]), we set to establish the feasibility of SPR analyses of the adaptive capability of a novel functional matrix encompassing AuDCFs formed from dynamic constitutional frameworks, DCFs with incorporated AuNP, when exposed to given external stimuli, such as pH changes.

### 1.4. Justification of the Selection of the Model

A tri-component mixture consisting of benzene-1,3,5-tricarbaldehyde (BTA) connection centres, and poly(ethylene glycol) bis(3-aminopropyl) terminated and thiol-poly(ethylene glycol)-amine (SH-PEG-NH2) connectors was chosen as the model adaptive material for the studies described here. Their self-assembly toward DCFs results in the formation of robust supramolecular motifs, which show spontaneous nucleation, interfacial control, and one-dimensional growth kinetics. These in turn lead to molecules stacking into fibres through π-π interactions and amide–amide hydrogen bonding, with these fibres forming a gelatinous network [23]. In parallel, PEGylated DCFs are characterized by higher flexibility—as related to PEG backbone—and multivalent proton-bonding via amide/amino functionalized entities—features that were found favourable for high enzyme (e.g., carbonic anhydrase) binding in solution [24] and even showed an enzyme activation effect towards carbonic anhydrase in solution.

In the current work, we combine imine based dynamic constitutional frameworks and AuNPs to design and synthesize double cross-linked Au-DCFs with multiple integrated functions. The combination of AuNPs, used as additional connecting centres, with dynamic DCFs, deployed as crosslinking components, offer the possibility to construct new nanoplatforms for the investigation of optical/electric properties under adaptive biomimetic conditions in response to a specific environment.

Accordingly, AuDCFs are proposed to be deposited on standard SPR chips via covalent immobilization. As test platforms, enabling assessment of the analytic effect of the location of the AuDCF versus the Au nanolayer of the SPR sensor, we envisage two types of sensing chips (Biacore C1 and Biacore CM5—https://www.cytivalifesciences.com, accessed on 8 November 2021) featuring surface linkers with different degrees of flexibility and volume versus surface binding. The DCFs conformational changes are analytically augmented when embedding AuNPs, which may play a double role: as sites for anchoring and labels for signal amplification.

Both experimental and theoretical analyses (consistent with the propagation of the evanescent field and SPR chip’s structure), complemented by Atomic Force Microscopy assays, were deployed to quantitatively assess and validate the adaptive response of the DCF to pH induced swelling or shrinkage upon increasing or decreasing the pH, respectively.

The originality of this work is based on reversible crosslinking points involved in previously unreported Au-DCFs structures and, moreover, on the very interesting chemical properties of the resulting materials used in pH sensing. This study provides also a completely unexplored insight into the sensing applicability of SPR real-time analysis of adaptive functional materials.

## 2. Materials and Methods

### 2.1. Materials

Common chemicals (NaCl, NaOH, Tween 20, ethylenediaminetetraacetic acid (EDTA), N-2-hydroxy-ethylpiperazine-N-2-ethane sulfonic acid (HEPES), 1-ethyl-3-(-3-dimethylaminopropyl) carbodiimide hydrochloride (EDC), N-hydroxysuccinimide (NHS), ethanolamine), HAuCl_4_·3H_2_O, sodium citrate, poly(ethylene glycol) bis(3-aminopropyl) terminated (PEG; 0.15 mmol, Mn~1500) and thiol-poly(ethylene glycol)-amine (SH-PEG-NH_2_; 0.05 mmol, Mn~2000) were purchased from Merck/Sigma-Aldrich (Darmstadt, Germany).

Benzene-1,3,5-tricarbaldehyde (BTA; 0.1 mmol) monomer was obtained from Manchester Organics (Runcorn, UK).

All aqueous solutions were prepared with ultrapure milliQ-water (Millipore, Burlington, MA, USA). HBS-EP (pH 7.4, 150 mM NaCl) was used as running buffer in all SPR experiments and was prepared according to the Biacore 3000 instrument manual (GE Life Sciences Solutions, Marlborough, MA, USA). Variable pH solutions (5–6.5) were prepared by adjusting the HBS-EP’s pH with aqueous NaOH (1 M). The pH of each solution was verified using an Inolab pH 720 (WTW, Weilheim, Germany) pH meter.

### 2.2. Gold NPs

AuNP were prepared by the well-known Turkevich method [25]. Briefly, 62 mg of HAuCl_4_·3H_2_O was dissolved in 50 mL of milliQ-water with stirring at 60 °C under reflux. Then, the reducing agent sodium citrate tribasic was injected. The mixture was incubated for 3 h under vigorous stirring at 85 °C. The solution turned red, indicating the formation of citrate-stabilized gold nanoparticles. The size of obtained particles was characterized by Dynamic Light Scattering (DLS) using a Malvern Zetasizer Nano-S ZEN1600 (Malvern Instruments, Malvern, UK) with a 173° backscatter measurement angle and a quartz cuvette with a square aperture. The morphology was observed by transmission electron microscopy (TEM) (JEM-1400+, Jeol Ltd., Tokyo, Japan) using a carbon 400 mesh copper grid, on which 10 µL of each sample were added and dried for 10 min.

### 2.3. Au-DCFs Synthesis

**Scheme 1:**
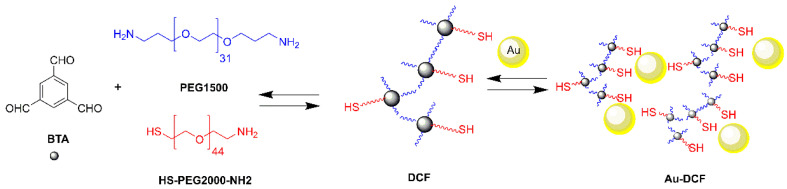
Synthesis route for Au-DCFs.

Preparation of DCFs: benzene-1,3,5-tricarbaldehyde (BTA; 0.1 mmol), poly(ethyleneglycol)-bis(3-aminopropyl)-terminated (PEG1500; 0.15 mmol, Mn~1500) and thiol-poly(ethylene glycol)-amine (SH-PEG2000-NH_2_; 0.05 mmol, Mn~2000), monomers were dissolved in MeOH, and the reaction mixture was kept stirring at 60 °C overnight. Then, the solvent was evaporated and replaced by milli-Q water to form a stock solution of 10 mM that is further used for Au-DCF synthesis according to Scheme 1.

Au-DCFs synthesis: 750 µL of 0.21 mM freshly prepared citrate-stabilized gold nanoparticles was mixed with BTA/PEG/SH-PEG-NH_2_ (1:1.5:0.5 molar ratio) DCF in an aqueous solution at different gold to DCF molar ratios 1/8, 1/10, 1/15. The mixture was stirred (200× *g* rpm) overnight at room temperature. The obtained Au-DCF network was characterized by DLS and TEM.

### 2.4. Sensor Chip Functionalization

Biacore sensor chips with carboxyl terminated surfaces (C1—planar carboxylated surface and CM5—carboxymethylated dextran surface, Cytiva, Marlborough, MA, USA) were used for covalent immobilization of Au-DCFs. The immobilization was performed in the Biacore 3000 external Surface Prep Unit (GE Healthcare Life Sciences Solutions, Marlborough, MA, USA) and the protocol consisted of: injection of EDC/NHS activation solution (200 mM EDC and 50 mM NHS in water) for 7 min, injection of Au-DCF (various gold to DCF molar ratio—experiment dependent) for 30 min at a flow rate of 2 μL·min^−1^ and injection of ethanolamine 7 min for deactivation of unreacted carboxylic groups.

### 2.5. Surface Plasmon Resonance Measurements

All SPR measurements were performed at 25 °C in a four flow-cell Biacore 3000 instrument (GE Healthcare Life Sciences Solutions, Marlborough, MA, USA) with HBS-EP, pH 7.4, 150 mM NaCl as running buffer (RB). Automatic, sequential injections were applied on different channels individually to limit sample dispersion effects (dilution of sample in RB at the beginning and end of the injection) and to avoid carry over between flow cells if Au-DCF dissociation occurs in response to pH changes. The duration of injection was set typically to 5 min to assess the short-term dynamic changes within the matrix and minimize the occurrence of irreversible changes.

Briefly, after a 5 min of baseline recording, HBS-EP solutions of different pHs were injected into the respective channels at a flow rate of 10 μL·min^−1^ for 5 min, followed by a HBS-EP wash-out step. The flow rate was optimized to avoid mass transport phenomena, as well as to minimize flow induced changes of the dynamer matrix. 

For baseline referencing, we marked a reference point 5 s prior to each injection. This value was subtracted from the signal values. Additionally, the signal recorded on the reference channel (not immobilized with Au-DCFs) was subtracted from the signal of the sample channels, thus applying a double-referencing procedure: correction of the device drift and of the refractive index difference between the running buffer and the injected solutions.

### 2.6. Complementary Characterization (AFM)

The AFM scan images were obtained in intermittent contact mode and in air using a Nanowizard II instrument from JPK Instruments A.G. (Berlin, Germany) on SPR chips (removed from their casing). The AFM images were obtained using line rates of 0.3 Hz and ARROW-NC AFM probes (from Nano World A.G., Neuchâtel, Switzerland), with cantilevers characterized by a resonance frequency around 160 kHz and a force constant of 7.2 N m^−1^). The ratio between the set-point amplitude and the free amplitude of the AFM cantilever was set to 0.5–0.6. Two separate images were made for each SPR channel.

### 2.7. Theoretical Framework

Theoretical simulations were based on the transfer matrix method, proven effective to reveal the effects of multilayer stacks analysed by SPR [26]. SPR chips (presenting 2D surface and 3D functional matrices and Au-DCFs) were simulated by considering a thin film of gold (50 nm thickness) deposited on glass (BK7) with an intermediate adhesion layer of titanium (2 nm). The model encompasses additional layers on top of the Au film for the functional matrix (in case of the CM5 chip) and the Au-DCFs on both type of chips (C1 and CM5). Refractive indexes values corresponding a wavelength of 760 nm (Biacore 3000 light source) for known materials (BK7 glass, Ti, Au) were obtained from refractiveindex.info online database, accessed on October 2021. The 3D functional matrix and Au-DCFs were added, taking in account their refractive indexes [27] and the AuNPs/DCF volumetric ratio (derived from TEM and AFM measurements).

## 3. Results and Discussion

### 3.1. Characterization of Au-DCFs

**Figure 1:**
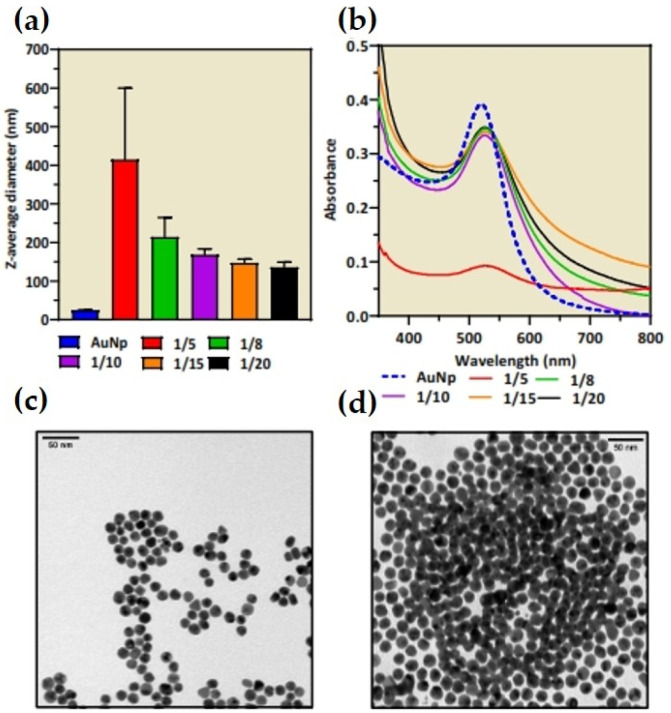
(**a**) Z-average diameter of gold nanoparticles and Au/dynamer conjugates determined by DLS (*n* = 3); (**b**) UV-Visible spectra of gold nanoparticles and Au- DCF. TEM images of (**c**) citrate-stabilized gold nanoparticles at 0.105 mM and (**d**) of Au-DCF conjugates at 1/20 Au/DCF molar ratio.

The size of AuNPs and Au-DCFs was determined by DLS. AuNPs of 19.5 ± 0.2 nm diameter, formed well-dispersed assemblies in the presence of DCFs in water (PDI < 0.5) with the size significantly increasing, the higher the Au/DCF ratios (see Figure 1a). The size of the assembly was related to the amount of dynamer added in a way that it reached a high value of 415 nm at 1/5 gold to dynamer molar ratio. Then, it decreased to lower values (200–150 nm) proportionally to the amount of dynamer. These conjugates were also characterized for the morphology by TEM. DLS assay revealed citrate-stabilized gold nanoparticles at 0.105 mM as individual 19 nm spherical particles, slightly aggregating and homogeneously distributed in solution. In comparison, TEM data showed slightly smaller particles, around 14 nm. In the presence of dynamer at 2.1 mM concentration, the mixture Au-DCFs formed highly aggregated clusters with DCFs networks connecting the particles. Due to dynamic restructuring, the morphology of the aggregates could vary in time, therefore larger aggregates could also be envisioned for all Au/DCF ratios.

Citrate stabilized AuNPs exhibited a surface plasmon resonance (SPR) at 520 nm; characteristic peak of Au(0) NPs [28]. The SPR peak was similar, before and after addition of DCF, in shape and width. The SPR peak of Au-DCFs at 1/5 molar ratio was broadened and with lower intensity compared to other ratios due to extensive aggregation. The peak absorbance of Au upon Au-DCF interaction, 1/8 to 1/20 Au to DCF molar ratios, was slightly decreased coupled to small redshifts in comparison with AuNP (Figure 1b). These observations proved that gold-thiol interactions were significant enough to stabilize the Au-DCFs network and maintain the optical properties of Au. DCF controlled the well-dispersion of Au-NPs at Au/DCF ratios above 1/5.

### 3.2. SPR Measurement of Au-DCF

**Figure 2:**
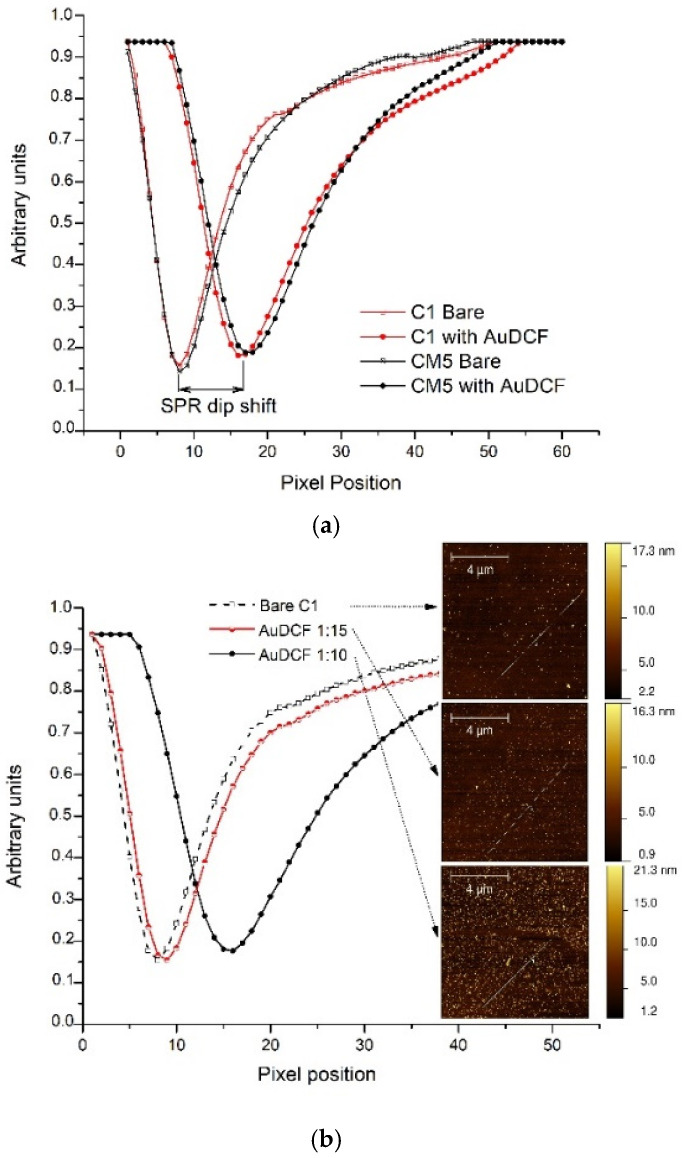
(**a**) SPR curves recorded on C1 and CM5 chips showing successful attachment of Au-DCFs; (**b**) SPR curves recorded on C1 for Au-DCFs with different gold to dynamer ratios—Inset AFM scans on each surface revealing Au-DCF aggregates on the corresponding surfaces.

Au-DCFs’ successful attachment, as a distinct matrix layer, on both types of investigated SPR chips was demonstrated by the increase in the SPR baseline values recorded prior and after immobilization: ~16,000 ΔRU for CM5 and ~15,400 ΔRU for C1 (RU—Biacore 3000 relative units 1 RU corresponding to 10^−6^ refractive index units and to an estimated 1 fg mm^−2^ of protein). We also recorded snapshots of whole SPR curves (not just the position of the minimum) for the two surfaces prior and after dynamer immobilization (Figure 2a). The attachment of the DCF matrix was reflected in widened SPR dips shifted towards higher values.

Two gold to DCF ratios were investigated by immobilization of the Au-DCFs (1:10 and 1:15) on different flow cells on the same chip. Higher dynamer amounts, with correspondingly larger clumps were not used to avoid clogging of the flow channels of the instrument, in case of detachment from the SPR surface. The influence of the ratio (shown in Figure 2b) is related to the absolute dynamer thickness evident as ~2600 RU ± 150 increase in the baseline value for the thin layer (1:15 red curve shifted from the bare C1) and ~15,400 RU ± 300 for the thick layer (1:10 black circles) versus the same bare C1 reference. Similar values were obtained for CM5 sensors (data not shown). Additional AFM images (Figure 2b inset) confirm the SPR data. AFM scans reveal a surface that is densely populated by particles in the case of Au-DCF with 1:10 ratio, fewer particles in the case of the 1:15 ratio and no particles in the reference channel. The large formations observed in the reference channel are due to reminiscences of buffer crystals inherent in flow measurements. The amplification effect provided by AuNPs onto the SPR signal (particularly on the θ_SPR_ shift) is due to the high contrast between the refractive index values of NPs versus the DCF or Buffer ones. Since DCF controlled the well-dispersion of Au-NPs, at ratios used in our study in a highly hydrated volume above the SPR surface, the large distance between AuNPs (over tens of nanometres—Figure 2 inset) limits their mutual interaction, as highlighted for example in our previous study [29]. Accordingly, addressing the mechanism behind signal augmentation due to use of AuNPs in the dynameric framework is not straightforwardly associated with the typical mechanisms related to presence of AuNPs on plasmonic surface, i.e., intensification of AuNP induced dipolar screening effects such as Debye screening effects or formation of hot spots between gold surface and AuNP deserves a study on its own.

### 3.3. Conformational Changes of the Au-DCFs Due to pH Changes Revealed by SPR Measurements

The stability of the DCFs to neutral and acidic pH was previously investigated. It was observed that the dynamers are stable at neutral and slightly acidic pHs > 3 (and remain unchanged for up to 7 days) and are strongly hydrolysed and become unstable in highly acidic medium (pH = 1) [12]. Additionally, previous baseline signal studies showed that the exposure of bare carboxymethyl-dextran (CMD) coated substrates, such as on the Biacore CM5 chips, to solutions of varying pH affects the baseline as an indicator of a structural change in the CMD. CMD surfaces are mostly affected by pHs below 1 and above 11 [18]. Having in mind the above observations, for our investigation we chose pHs in the range 5.5–7.4 that do not induce the degradation of the dynamer and potentially do not affect the chip surface.

As previously investigated [19], linear biomacromolecules show conformational changes with subsequent swelling/shrinkage by undergoing intramolecular (e.g., electrostatic) interactions in response to pH changes in the surrounding media.

Theoretical simulations, based on the transfer matrix method, showed a variation of the SPR dip position in respect to the type of the chip (2D planar surface and 3D matrix) and thickness modification of the added layer. The Au-DCF layer was simulated containing AuNP of 20 nm and distance between adjacent AuNPs of 100 nm (values provided by DLS, and AFM measurements). The refractive index of the AuNP and DCF mixture forming the Au-DCF was calculated using the Arago-Biot relation [30]. Aiming to assess DCF structural changes, several concentrations of DCF or DCF and AuNPs in buffer were considered (i.e., the volumetric ratios: V_DCF_/V_total_ = 0.37; V_AuNPs_/V_total_ = 0.1 and V_Buffer_/V_total_ = 0.62 and 10% decrease and successively 10% increase of the volumetric ratio of AuNPs and DCF mixture, keeping the same ratio V_AuNPs_/V_DCF_ of the individual components) to simulate for either expansion or contraction of the framework following exposure to pH step changes.

**Figure 3:**
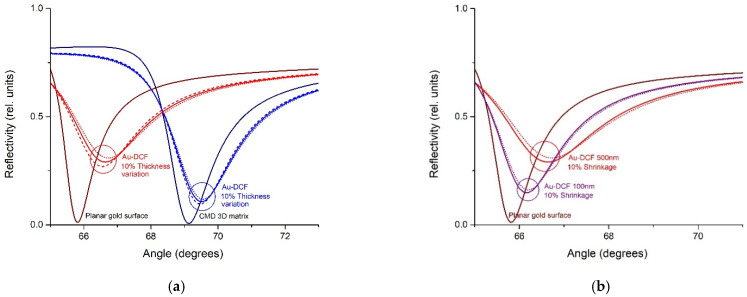
(**a**) Theoretical SPR curves on planar gold surface (dark red) and CMD-coated gold surface (dark blue); the addition of Au-DCF shifts the SPR curve minimum to higher angles: coated planar gold surface (continuous red line), coated CMD (continuous blue line); dotted and dashed lines show the effect of the ±10% variation of the thickness of dynamer; (**b**) Theoretical SPR curves on planar gold surface (dark red) covered with a layer of 100 nm Au-DCF (purple) and 500 nm Au-DCF (red). The effect of 10% contraction of the Au-DCF is shown in dotted lines.

Simulations performed on DCFs with and without AuNP reveal a greater effect of matrix conformational changes when AuNP are present. Furthermore, the simulations reveal the expected differences in the SPR response to the binding of the same polymeric matrix, encompassing AuNP (with height beyond the penetration depth of the evanescent field) when the matrix is bound almost directly on the Au interface of the SPR sensors versus the case when binding occurs on top of a dextran layer of ~150 nm. As shown in Figure 3a, the same polymeric matrix provides a significantly larger SPR response (shifts in the SPR curve minimum) when the binding occurs in the immediate vicinity of the plasmonic surface. 

Volume variations, shrinkage/extension, were simulated by considering a constant volumetric ratio of AuNP vs. DCF matrix while changing the volume ratio of the Au-DCFs and the buffer. The influence of Au-DCF layer thickness to the structure of the SPR signal was simulated by considering the Au-DCF matrix having thicknesses of 100 nm and 500 nm, respectively. It is shown in Figure 3b that the thicker the Au-DCF, the greater the effect of matrix shrinkage on the SPR signal. Simulations pertaining to the effect of the AuNP on the SPR signal are presented in the Supplementary Materials (Figure S1).

**Figure 4:**
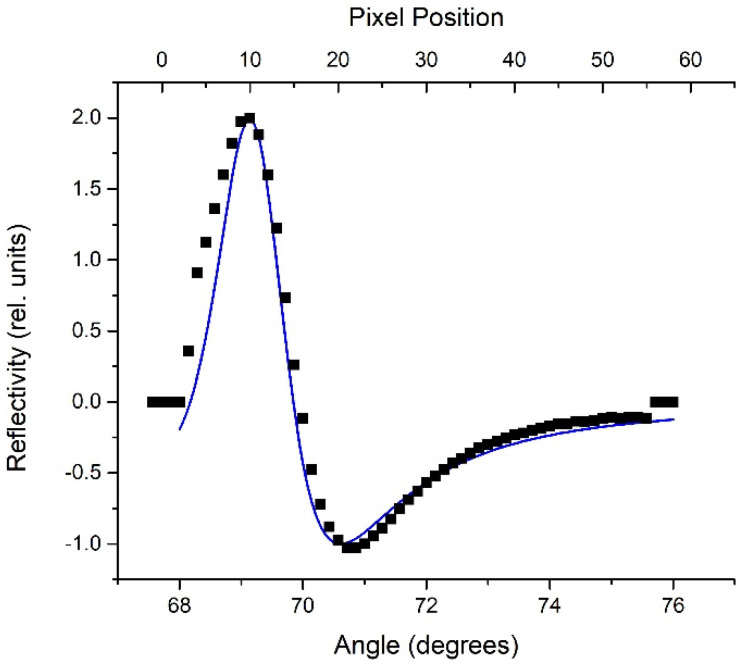
Normalized difference between theoretical SPR curves of the same layer with ±10% variation of thickness (blue line) and normalized difference between measured SPR curves at pH 7.4 and 6 on a C1 SPR chip (black points).

The SPR signal reflects dynamic changes within the Au-DCF matrix covalently bound to a plasmonic chip upon step changes of 2 units pH (from 7.4 to 5.5) highlighting the analytical potential of the Au-DCF matrix. The SPR signals (referenced only for the baseline drift—i.e., signal vs. a baseline reference point) reveal changes in the Au-DCF conformation in comparison with the reference channel. These are visible as a signal increase (refractive index increment) that exceeds the effect of the changing refractive index of the buffer. As shown in Figure 4, the simulations are consistent with the experimental data, as the normalized difference between theoretical SPR curves of the same layer with different thicknesses is similar with the one obtained by calculating the difference between SPR curves measured at two different pHs (7.4 and 6). This indicates that decreases in pH trigger the shrinkage of the matrix (increased concentration of DCF and AuNP components).

**Figure 5:**
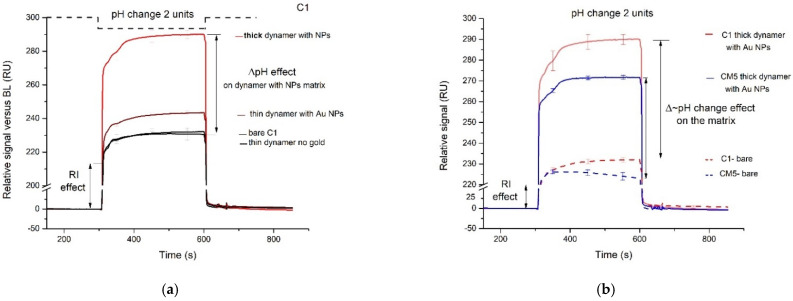
SPR signal of plasmonic chips with immobilized Au-DCFs, covalently bound to the surface, upon pH step changes from 7.4 to 5.5: (**a**) The effect of the Au-DCF thickness (**b**) The effect of the supporting functional layer—CM5 vs. C1 chips. pH change 2 units means passing HBS-EP buffer solution of pH 5.5 (instead of the buffer solution of pH 7.4) through the measurement chamber, for the indicated duration.

The SPR signals are specifically related to the thickness of the Au-DCFs. For the same pH variation, we quantified a signal, due mostly to RI change in the bare C1 surface, a slight increase for the thin Au-DCF (1:15) and, correspondingly, a much higher response for the thick Au-DCF (Figure 5a). Additionally, the effect of the pH change on a DCF matrix without gold is very low, similar to the bare C1 surface, revealing the importance of the presence of AuNP in the DCFs. The interaction of DCF with the surface of AuNPs is confirmed using IR spectroscopy analysis (Figure S2) Supplementary Materials.

As predicted, larger responses expected when the dynamic changes take place closer to the plasmonic chip, are experimentally demonstrated according to the comparison between C1 and CM5, presented in Figure 5b. CM5 chips present an intermediary ~150 nm CMD matrix as a substrate for the Au-DCF, while the C1 chip is only covered with a planar carboxylated layer.

**Figure 6:**
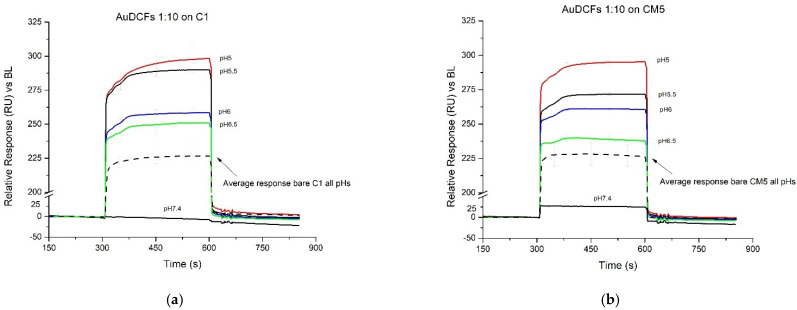
SPR sensorgrams of different pH solution injections on Au-DCF 1:10 covalently immobilized on: (**a**) C1 chip and (**b**) CM5 chip. The black dotted line shows the average response of the blank channel on each chip, to all pH solutions.

Having established that SPR assay is sensitive to conformational changes in the Au-DCFs, we set to reveal the SPR signature of DCF response to changing pHs. We characterized the response of the Au-DCFs covered SPR chips to different pH by programming the instrument to perform a series of 5 replicates of injections of each pH. SPR sensorgrams of the individual injections of HBS-EP buffer (same as the running buffer), with adjusted pH values of 5, 5.5, 6, 6.5 and 7.4 (as control) are presented in Figure 6.

A larger response is achieved when the dynamic changes take place closer to the plasmonic chip (comparison C1—Figure 6a and CM5—Figure 6b). Greater variability is evident for C1 chips, possibly due to the larger sensitivity on the Au-DCF heterogeneity, their complex matrix being closer to the sensing surface. Due to the limited flexibility of DCF or DCF and AuNPs provided by the planar C1 sensing chips, larger responses (when using C1 versus CM5 sensing chips) were noticed for a limited range of pH step changes (7.4–5.4).

**Figure 7:**
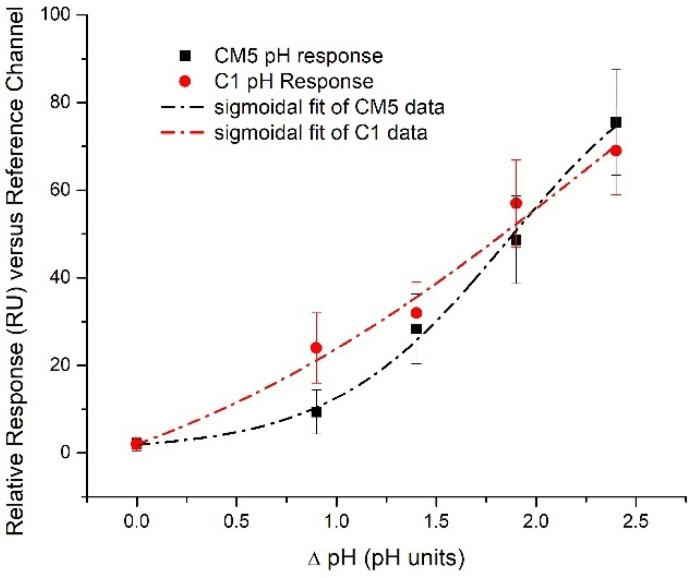
SPR response dependence on pH step changes (towards acidic domain) of the same Au-DCFs covalently immobilized on C1 (red) and CM5 (black) surfaces. Data is referenced versus the blank channel (without the Au-DCF) on each type of chips.

SPR response dependencies on pH step changes, towards acidic domain, (Figure 7), established following the experimental analyses, reveal different behaviour of the SPR differential signal (against the reference channel) in the case of the C1 planar surface versus the CM5 3D matrix. This behaviour is related to differences in the thickness of the supporting functional layer of the two types of chips. This aspect is well supported by both theory and experiment, since the same pH shift triggers a different SPR response, depending on the distance between the Au surface and the DCF. Consistent with Figure 6, it is observed that the same pH induced variation of the Au-DCF structure renders a slightly smaller SPR response for moderate pH steps (i.e., below 2 pH units) in the case of CM5, as compared to C1.

## 4. Conclusions

Dynamic constitutional frameworks augmented with embedded gold nanoparticles (i.e., Au-DCFs) were successfully prepared and investigated using a SPR assay. Au-DCFs were immobilized on two different types of functional chips to assess the influence of the supporting matrix to DCF mobility and its conformational reorganization under pH step changes.

The SPR data of both CM5 and C1 sensing chips are consistent with a reversible response, i.e., conformational reorganization of the constitutional framework (DCF and AuNP) in the pH range 5 ÷ 7.4, relevant for biosensing field.

Simulations consistent with experimental data indicate that the pH decrease from neutral to acidic pHs triggers a shrinkage of the DCF and AuNP matrix that can be dynamically monitored using SPR.

The thickness of the Au-DCFs layer, their Au content, as well as the distance from the sensing SPR surface, were demonstrated relevant for assay sensitivity. Thicker DCF layers showed increased response to pH relative to the thinner layers mediated by pH dependent conformational reorganization, irrespective of the type of SPR sensing surface used. However, as the pH triggered shrinkage is consistent with an increased volume concentration of Au-DCF components in the depth region relevant for SPR sensitivity, the chip structure modulates the assay sensitivity. The pH–SPR response of the CM5 and, respectively, C1 chips, encompass the combined effects of the differences between the 3D and 2D support of the functional layer of each chip. CM5 chips present a ~150 nm thick dextran matrix as a substrate for Au-DCF while the C1 chips have a planar distribution of the binding sites. Consistently, for the same variation of pH values, the matrix (DCF and AuNPs) structure more distant from the plasmonic surface (i.e., on CM5) renders a smaller SPR response, recommending planar plasmonic surfaces as best platforms for analysis. The apparent shift in sensitivity for pH steps above 2 units in favour of CM5 surfaces is related to a larger amount of DCF and AuNPs immobilized on CM5 surfaces, and to the increased flexibility of the dextran matrix, accommodating larger volume variations of the DCF and AuNPs matrix when triggered by exposure to lower pH values. Distinct pH response of the dextran matrix is also possible, thus CM5 use should be considered with care. This study reveals for the first time the SPR response, augmented by appropriate chip configuration, AuNPs presence and covalent immobilization strategy, to the conformational, i.e., shrinkage, change in a DCF matrix when subjected to pH drops, and provides new insights into the sensing applicability of SPR real-time analysis of adaptive functional materials. More generally, the results presented here show that using SPR chips functionalised with Au-DCF makes it possible to assess structural changes (expansion/contraction) on a nanometric scale, to radically transform sensing by deploying novel adaptive materials. For example, enzyme-loaded Au-DCFs could extend the use of SPR to the quantification of small molecular weight enzyme substrates, given the conversion of the substrate by the enzyme is accompanied by a pH change.

Whereas actual detection instances exceed the scope of our manuscript, the Au-DCF layer we are proposing is capable of reliably extending SPR-based detection to molecular targets whose enzymatic conversion causes pH variations, such as, for example, the detection of glucose (when it is modified with glucose oxidase), of urea (when it is modified with urease), and of penicillin when it is modified with penicillinase. The Au-DCF layer can also facilitate the SPR-based detection of molecules which inhibit an enzyme that catalyses a reaction causing pH variations. For example, it can facilitate the detection of acetazolamide when it is modified with carbonic anhydrase.

## Data Availability

Data generated during the present study are available from the corresponding authors on reasonable request.

## Supplementary Materials

The following supporting information can be downloaded at: https://www.mdpi.com/article/10.3390/ma15020483/s1, Figure S1: Theoretical SPR curves on bare gold surface (black), bare gold with a monolayer of AuNP (red), bare gold with a layer of DCF (blue), bare gold with a layer of AuDCF; Figure S2: FT-IR spectra of Au-NPs (black), DCF (blue) and Au-DCF network (green) at 1/20 gold to DCF molar ratio.
