# Supplementary material for: Real Time SPR Assessment of the Structural Changes of Adaptive Dynamic Constitutional Frameworks as a New Route for Sensing
Source: Materials (Basel). 2022 Jan 9;15(2):483. doi: 10.3390/ma15020483 (PMC8779961; doi:10.3390/ma15020483)
Supplement: Supplementary file 1 [file materials-15-00483-s001.zip › materials-1521519-supplementary.pdf]

Real time SPR assessment of the structural changes of adaptive dynamic constitutional frameworks as a new route for sensing

Sorin David [1 #], Mihaela Gheorghiu [1#], Sanaa Daakour [2], Raluca-Elena Munteanu [1], Cristina Polonschii [1], Szilveszter Gáspar [1], Mihail Barboiu [2*] and Eugen Gheorghiu [1*]

[1] International Centre of Biodynamics, 060101 Bucharest, Romania
[2] Institut Europeen des Membranes, Adaptive Supramolecular Nanosystems Group, University of Montpellier, ENSCM-CNRS, 34095 Montpellier, France
[#] Equal contribution
* Corresponding authors: mihail-dumitru.barboiu@umontpellier.fr and egheorghiu@biodyn.ro

-Supplementary material-

Simulation results (using Transfer matrix approach)

The SPR simulation results (using Transfer matrix approach) provided by AuNPs onto the surface of the sensing chip, without DCFs, are presented in Figure S1. It is shown that the addition of a AuNP monolayer the SPR dip is shifted towards lower angles due to the modification of the refractive index in the vicinity of the sensing gold layer. The explanation is that the refractive index of the liquid (i.e. water) is replaced with the refractive index of the AuNp which has a negative value. Adding the polymer shifts the SPR dip towards higher angles because the refractive index of the organic layer is higher than the liquid. The addition of a polymer containing AuNP leads to a SPR dip shift due to the combined effect of both constituents.

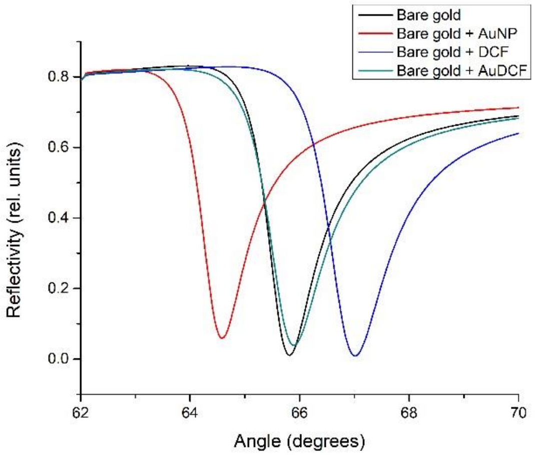

Figure S1. Theoretical SPR curves on bare gold surface (black), bare gold with a monolayer of AuNP (red), bare gold with a layer of DCF (blue), bare gold with a layer of AuDCF.

IR spectroscopy analysis:

Nicolet Nexus Fourier-transform infrared (FT-IR) spectrometer, equipped with ATR diamond Golden Gate, was used to show DCF interactions with the surface of AuNPs. AuNPs, DCF and Au-DCF networks, obtained as powders after lyophilization, were analyzed separately.

The IR spectrum of AuNP (Figure S2) shows strong absorption bands at 3362.4 cm^{-1} and 1388 cm^{-1} related to O-H bond of citrate ions on their surface [1]. The intensity of this band decreased significantly after addition of DCF (broaden peak around 3461.2 cm^{-1}). Thus, the surface properties of Au-NPs were modified with DCF interactions. The characteristic band of DCF -S-H stretching at 2741.2 cm^{-1}- disappeared in Au-DCF network spectrum, reminiscent with the high thiol binding on the surface of gold nanoparticles. The stretching bands: N-H (2882.4 cm^{-1}) and C=N (1696.7 cm^{-1}) are attributed to characteristic amino groups and imine bonds in DCF, respectively. Similarly in Au-DCF network N-H (2881.1 cm^{-1}) and C=N (1696.1 cm^{-1}). The dynamic structure of DCF was stable [2] in presence of gold nanoparticles.

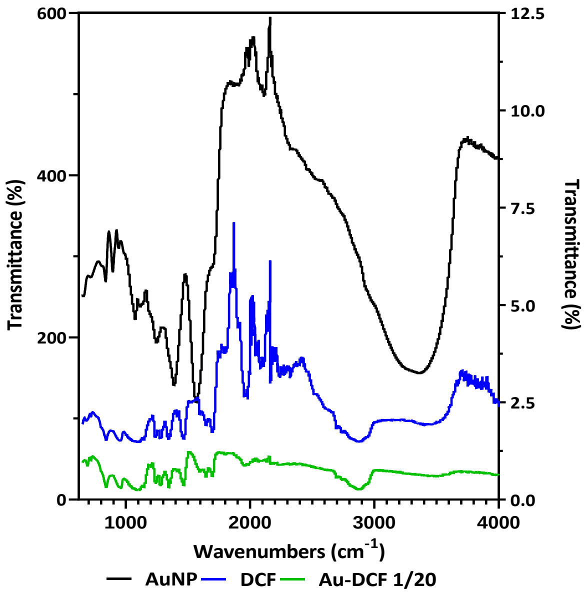

Figure S2. FT-IR spectra of Au-NPs (black), DCF (blue) and Au-DCF network (green) at 1/20 gold to DCF molar ratio.

Additional peaks, worth observing in the FT-IR spectra, are:

The band in 1466 cm^{-1} is assigned to aromatic C-C stretching vibrations of BTA.

The band around 828 cm^{-1} corresponds to the C-H stretching vibration of the benzene ring.

The strong absorption band at 1102.3 corresponds to C-O stretching of aliphatic ethers PEG

The IR spectrum of DCF showed a band at 1979.6 cm^{-1} (1650-2000 C-H bending of aromatic compound weak) that was absent in Au-DCF.

References

[1] L. Biao *et al.*, “Green Synthesis, Characterization and Application of Proanthocyanidins-Functionalized Gold Nanoparticles,” *Nanomaterials*, vol. 8, no. 1, Art. no. 1, Jan. 2018, doi: 10.3390/nano8010053.

[2] S. Karmakar, N. Sarkar, and L. M. Pandey, “Proline functionalized gold nanoparticles modulates lysozyme fibrillation,” *Colloids and Surfaces B: Biointerfaces*, vol. 174, pp. 401–408, 2019.
